# New Isocoumarin Derivatives and Meroterpenoids from the Marine Sponge-Associated Fungus *Aspergillus similanensis* sp. nov. KUFA 0013

**DOI:** 10.3390/md12105160

**Published:** 2014-10-13

**Authors:** Chadaporn Prompanya, Tida Dethoup, Lucinda J. Bessa, Madalena M. M. Pinto, Luís Gales, Paulo M. Costa, Artur M. S. Silva, Anake Kijjoa

**Affiliations:** 1 ICBAS—Instituto de Ciências Biomédicas de Abel Salazar, Universidade do Porto, Rua de Jorge Viterbo Ferreira 228, 4050-313 Porto, Portugal; E-Mails: chadaporn@buu.ac.th (C.P.); lbessa@ciimar.up.pt (L.J.B.); lgales@ibmc.up.pt (L.G.); pmcosta@icbas.up.pt (P.M.C.); 2 Interdisciplinary Centre of Marine and Environmental Research (CIIMAR), Rua dos Bragas 289, 4050-123 Porto, Portugal; 3 Department of Plant Pathology, Faculty of Agriculture, Kasetsart University, Bangkok 10240, Thailand; E-Mail: agrtdd@ku.ac.th; 4 Centro de Química Medicinal da Universidade do Porto (CEQUIMED-UP) and Laboratório de Química Orgânica e Farmacêutica, Departamento de Ciências Químicas, Faculdade de Farmácia, Universidade do Porto, Rua de Jorge Viterbo 228, 4050-313 Porto, Portugal; E-Mail: madalena@ff.up.pt; 5 Instituto de Biologia Celular e Molecular (IBMC), Universidade do Porto, 4099-003 Porto, Portugal; 6 Departamento de Química & QOPNA, Universidade de Aveiro, 4810-193 Aveiro, Portugal; E-Mail: artur.silva@ua.pt

**Keywords:** *Aspergillus similanensis*, similanpyrones, isocoumarins, meroditerpenes, pyripyropenes, chevalones

## Abstract

Two new isocoumarin derivatives, including a new 5-hydroxy-8-methyl-2*H*, 6*H*-pyrano[3,4-*g*]chromen-2,6-dione (**1**) and 6,8-dihydroxy-3,7-dimethylisocoumarin (**2b**), a new chevalone derivative, named chevalone E (**3**), and a new natural product pyripyropene S (**6**) were isolated together with 6, 8-dihydroxy-3-methylisocoumarin (**2a**), reticulol (**2c**), *p*-hydroxybenzaldehyde, chevalone B, chevalone C, S14-95 (**4**), and pyripyropene E (**5**) from the ethyl acetate extract of the undescribed marine sponge-associated fungus *Aspergillus similanensis* KUFA 0013. The structures of the new compounds were established based on 1D and 2D NMR spectral analysis, and in the case of compound **3**, X-ray analysis was used to confirm its structure and the absolute configuration of its stereogenic carbons. Compounds **1**, **2a**–**c** and **3**–**6** were evaluated for their antimicrobial activity against Gram-positive and Gram-negative bacteria, *Candida albicans* ATCC 10231, and multidrug-resistant isolates from the environment. Chevalone E (**3**) was found to show synergism with the antibiotic oxacillin against methicillin-resistant *Staphylococcus aureus* (MRSA).

## 1. Introduction

**Figure 1:**
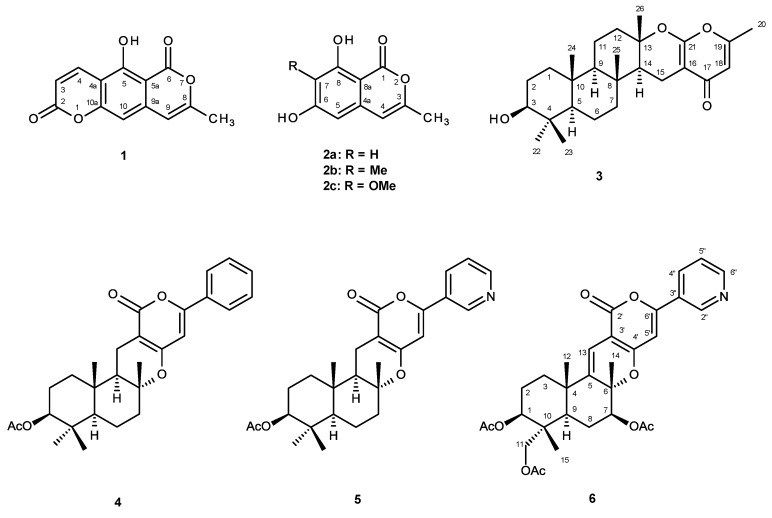
Secondary metabolites from *Aspergillus similanensis* KUFA0013.

*Neosartorya* is a teleomorphic (sexual) state of *Aspergillus* section *Fumigati*. Although *Neosartorya* species (Trichocomaceae) have not been as extensively investigated for their secondary metabolites as *Aspergillus*, they have recently been shown to be an interesting source of many bioactive compounds [1,2,3,4,5,6,7,8,9]. In our ongoing search for new natural products with antibacterial activity produced by the marine-derived fungi of the genus *Neosartorya*, we have investigated the secondary metabolites of a Thai collection of a new species of *Neosartorya*, isolated from the marine sponge *Rhabdermia* sp., collected from the Similan Islands, Phang Nga Province, Southern Thailand. However, in order to comply with the recent “International Code of Nomenclature for algae, fungi and plants (The Melbourne Code)”, the strain was renamed *Aspergillus similanensis* (KUFA0013). The ethyl acetate extract of its culture furnished, besides chevalones B and C [9,10], *p*-hydroxybenzaldehyde, reticulol (**2c**) [11], 6,8-dihydroxy-3-methylisocoumarin (**2a**) [12], a meroterpenoid S14-95 (**4**) [13], pyripyropene E (**5**) [14], two new isocoumarins which we have named similanpyrones A (**1**) and B (**2b**), a new chevalone analog (**3**), and a new natural product which we have named pyripyropene S (**6**) [15] (Figure 1). Compounds **1**, **2a**–**c**, **3**–**6** were evaluated for their antimicrobial activity against Gram positive (*Staphylococcus aureus* ATCC 25923 and *Bacillus subtilis* ATCC 6633) and Gram negative (*Escherichia coli* ATCC 25922 and *Pseudomonas aeruginosa* ATCC 27853) bacteria, *Candida albicans* ATCC 10231, as well as multidrug-resistant isolates from the environment.

## 2. Results and Discussion

Compound **1** was isolated as white solid (mp, 322–323 °C), and its molecular formula C_13_H_8_O_5_ was established on the basis of the (+)-HRESIMS *m/z* 245.0450 [M + H]^+^, indicating ten degrees of unsaturation. The IR spectrum showed absorption bands for hydroxyl (3446 cm^−1^), conjugated lactone carbonyl (1748, 1698 cm^−1^), aromatic (1658 cm^−1^) and olefin (1634, 1464 cm^−1^) groups. The ^13^C NMR (Supplementary Information, Figure S2), DEPTs and HSQC spectra (Table 1, Supplementary Information, Figure S4) revealed the presence of two conjugated ester carbonyls (δ_C_ 166.3 and 159.7), six quaternary sp^2^ (δ_C_ 101.3, 107.3, 130.0, 140.1, 156.2 and 160.3), four methine sp^2^ (δ_C_ 102.7, 104.6, 114.1, and 137.8) and one methyl (δ_C_ 19.6) carbons. The ^1^H NMR spectrum (Supplementary Information, Figure S1) revealed, besides a singlet of the hydrogen bonded hydroxyl proton at δ_H_ 11.90, two doublets of the *cis*-olefinic protons at δ_H_ 8.13 (*J* = 9.8 Hz) and 6.36 (*J* = 9.8 Hz), two singlets at δ_H_ 6.33 and 6.70, and one methyl singlet at δ_H_ 2.33. The COSY spectrum (Table 1; Supplementary Information, Figure S3) exhibited cross peak between the singlet at δ_H_ 6.33 (H-9) and the methyl singlet at δ_H_ 2.33 (Me-8), suggesting that they were allylically coupled. On the other hand, the HMBC spectrum (Table 1; Supplementary Information, Figure S5) showed cross peaks of H-9 to C-8 (δ_C_ 156.2), C-9a (δ_C_ 130.3), C-10 (δ_C_ 102.7), and C-5a (δ_C_ 101.3), of H-10 (δ_H_ 6.70, s) to C-5a, C-4a (δ_C_ 107.3), C-9a and C-10a (δ_C_ 140.1), of Me-8 to C-8 and C-9 (δ_C_ 104.6), and of OH-5 (δ_H_ 11.90) to C-5 (δ_C_ 160.3), C-5a and C-4a. Taking together the ^1^H and ^13^C chemical shift values and the COSY, HSQC and HMBC correlations (Table 1), the presence of 4a, 10a-disubstituted-5-hydroxy-8-methylisochromen-6-one was corroborated. That the 5-hydroxy-8-methylisochromen-6-one nucleus was fused with a pyran-2-one moiety on C-4a and C-10a was substantiated by the HMBC correlations of H-4 (δ_H_ 8.13, d, *J* = 9.8 Hz) to C-10a (δ_C_ 140.1), and of H-3 (δ_H_ 6.36, d, *J* = 9.8 Hz) to C-4a (δ_C_ 107.3) and C-2 (δ_C_ 159.7), respectively. Thus, the structure of compound **1** was established as 5-hydroxy-8-methyl-2*H*, 6*H*-pyrano[3,4-g]chromene-2,6-dione. To the best of our knowledge, this is the first report on the isolation of a secondary metabolite with both coumarin and isocoumarin functionalities in the same molecule. Thus, compound **1** is a new compound which we have named similanpyrone A.

**Table 1:** ^1^H and ^13^C NMR (CDCl_3_, 500.13 MHz and 125.8 MHz) and HMBC assignment for similanpyrone A (**1**).

Position	δ_C_, Type	δ_H_, (*J* in Hz)	COSY	HMBC
				
2	159.7, C	-		
3	114.1, CH	6.36, d (9.8)	H-4	10a
4	137.8, CH	8.13, d (9.8)	H-3	C-2, 4a
4a	107.3, C	-		
5	160.3, C	-		
5a	101.3, C	-		
6	166.3, C	-		
8	156.2, C	-		
9	104.6, CH	6.33, s	CH_3_-8	C-5a, 8, 10, Me-8
9a	130.0, C	-		
10	102.7, CH	6.70, s		C-4a, 5a, 9a, 10a
10a	140.1, C	-		
CH_3_-8	19.6, CH_3_	2.33, s	H-9	C-8, 9
OH-5	-	11.90, s		C-4a, 5, 5a

Compound **2b** was also isolated as white solid (mp, 162–163 °C), and its molecular formula C_11_H_10_O_4_ was established on the basis of the (+)-HRESIMS *m/z* 207.0658 [M + H]^+^, indicating seven degrees of unsaturation. The IR spectrum showed absorption bands for hydroxyl (3243, 3160 cm^−1^), conjugated carbonyl (1677 cm^−1^), olefin (1635 cm^−1^) and aromatic (1617 cm^−1^) groups. The general feature of the ^1^H (Supplementary Information, Figure S6), and ^13^C NMR spectra (Supplementary Information, Figure S7) of **2b** (Table 2) closely resembled those of **1**, except for the absence of the proton and carbon signals of the pyran-2-one moiety. Instead, there were an additional methyl (δ_H_ 2.00 s; δ_C_ 8.0) and hydroxyl (δ_H_ 3.45 brs) groups in the structure of **2b**. That the second methyl group was on C-7 and the second hydroxyl group was on C-6 was corroborated by the HMBC cross peaks of the methyl singlet at δ_H_ 2.20, s to the signals of C-6 (δ_C_ 163.7), C-7 (δ_C_ 109.6) and C-8 (δ_C_ 106.9). Thus, the structure of compound **2b** was established as 6, 8-dihydroxy-3, 7-dimethylisochromen-1-one. Literature survey revealed that **2b** is a new compound, and therefore we have named it similanpyrone B.

**Figure 2:**
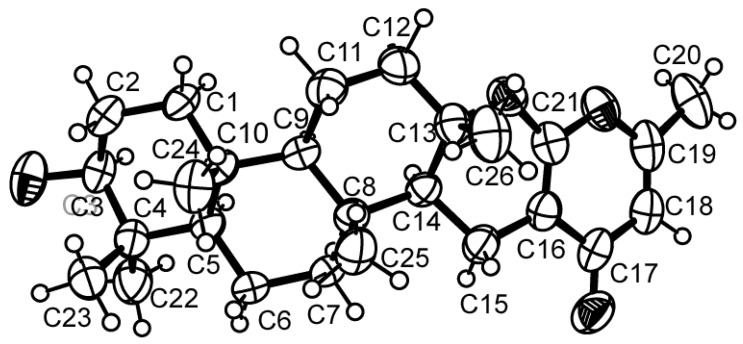
Ortep view of chevalone E (**3**).

Compound **3** was isolated as white crystals (mp, 262–263 °C), and its molecular formula C_26_H_38_O_4_ was established on the basis of the (+)-HRESIMS *m/z* 415.2851 [M + H]^+^, indicating eight degrees of unsaturation. The IR spectrum showed absorption bands for hydroxyl (3300 cm^−1^), conjugated carbonyl (1664 cm^−1^) and olefin (1607, 1570 cm^−1^) groups. The ^13^C NMR (Supplementary Information, Figure S9), DEPTs and HSQC spectra revealed the presence of one conjugated ketone carbonyl (δ_C_ 180.6), three quaternary sp^2^ (δ_C_ 162.6, 160.5, 98.5), one methine sp^2^ (δ_C_ 111.9), one oxygen bearing quaternary sp^3^ (δ_C_ 84.3), one oxygen bearing methine sp^3^ (δ_C_ 78.7), three quaternary sp^3^ (δ_C_ 37.1, 37.3, 38.9), three methine sp^3^ (δ_C_ 52.3, 55.3, 60.3), seven methylene sp^3^ (δ_C_ 15.2, 17.9, 18.7, 27.2, 38.4, 40.1, 41.1) and six methyl (δ_C_ 15.4, 16.1, 16.4, 19.2, 20.5, 28.0) carbons. The general feature of the ^1^H (Supplementary Information, Figure S8), and ^13^C NMR spectra of **3** resembled those of chevalone C [9], except for the chemical shift values of the oxygen bearing methine carbon (C-3) which appeared at lower frequencies (δ_C_ 78.7; δ_H_ 3.21, dd, *J* = 11.1, 5.0 Hz) than those of chevalone C [9]. Furthermore, the ^1^H and ^13^C NMR spectra of compound **3** did not exhibit the signals of the acetyl group. Taking together the IR, HRMS and NMR data, it was possible to conclude that compound **3** is a deacetyl analog of chevalone C. Since this is the first report of isolation of this chevalone analog, we have named it chevalone E. Final proof of the structure and the stereochemistry assigned to chevalone E (**3**) was provided by an X-ray analysis (Figure 2), and since the diffraction data were collected with a Gemini PX Ultra equipped with CuKα radiation, it was possible to establish the absolute configuration of C-3, C-5, C-8, C-9, C-10, C-13 and C-14, respectively as 3*S*, 5*R*, 8*R*, 9*R*, 10*R*, 13*S* and 14*S*.

**Table 2:** ^1^H and ^13^C NMR (CDCl_3_, 500.13 MHz and 125.8 MHz) and HMBC assignment for similanpyrone B (**2b**).

Position	δ_C_, Type	δ_H_, (*J* in Hz)	COSY	HMBC
1	166.1, CO	-		
3	153.3, C	-		
4	104.2, CH	6.46, s	CH_3_-3	C-5, 8a
4a	136.5, C	-		
5	101.4, CH	6.40, s		C-4, 6, 7, 8a
6	163.7, C	-		
7	109.6, C	-		
8	160.0, C	-		
8a	97.5, C	-		
CH_3_-3	18.8, CH_3_	2.20, s		C-3, 4
CH_3_-7	8.0, CH_3_	2.00, s		C-6, 7, 8
OH-6	-	3.45, br		
OH-8	-	11.27, s		C-7, 8, 8a

The (+)-HRESIMS of compound **6** indicated the [M + H]^+^ peak at *m/z* 566.2416, corresponding to C_31_H_36_NO_9_. Thus, the molecular formula of compound **6** was C_31_H_35_NO_9_, indicating fifteen degrees of unsaturation. The IR spectrum showed absorption bands for ester carbonyl (1742 cm^−1^), conjugated carbonyl (1671 cm^−1^), aromatic (1586, 1508, 1465 cm^−1^) and olefin (1625 cm^−1^) groups. The ^13^C NMR (Supplementary Information, Figure S11), DEPTs and HSQC spectra (Table 3) revealed the presence of three ester carbonyls (δ_C_ 171.0, 170.4, 169.8), one conjugated carbonyl (δ_C_ 161.3), five quaternary sp^2^ (δ_C_ 161.2, 157.2, 144.5, 127.4, 101.1), six methine sp^2^ (δ_C_ 152.1, 146.6, 133.1, 123.8, 111.2, 98.6), one oxyquaternary sp^3^ (δ_C_ 83.9), two oxymethine sp^3^ (δ_C_ 77.7 and 73.2), one oxymethylene sp^3^ (δ_C_ 64.7), two quaternary sp^3^ (δ_C_ 40.6 and 38.8), one methine sp^3^ (δ_C_ 41.1), three methylene sp^3^ (δ_C_ 35.5, 24.3 and 23.2), and six methyl (δ_C_ 24.2, 21.3, 21.2, 21.2, 20.8 and 13.3) carbons. Analysis of the ^1^H (Supplementary Information, Figure S10), ^13^C, HSQC and HMBC spectra (Table 3) revealed the presence of, besides three acetoxyl groups (δ_C_ 170.4, 21.2, δ_H_ 2.05s; δ_C_ 171.0, 20.8, δ_H_ 2.10s; δ_C_ 169.8, 21.2, δ_H_ 2.17, s), a hexasubstituted decahydronaphthalene ring system. That two of the acetoxyl groups were on C-1 and C-7, and the three methyl groups were on C-4, C-6 and C-10 of the decahydronaphthalene moiety was substantiated by the HMBC cross peaks of the Me-15 singlet (δ_H_ 0.88, s) to the carbon signals at δ_C_ 40.6 (C-10), 72.2 (C-1), 64.7 (C-11), of the Me-12 singlet (δ_H_ 1.26, s) to the carbon signals at δ_C_ 35.5 (C-3), 38.8 (C-4), 41.1 (C-9), and of the Me-14 singlet (δ_H_ 1.59, s) to the carbon signals at δ_C_ 77.7 (C-7), 83.9 (C-6), and 144.5 (C-5). That another substituent on C-10 was the acetoxymethylene group was evidenced by the HMBC cross peaks of H-11 signals (δ_H_ 3.75, d, *J* = 11.9 Hz; 3.79, d, *J* = 11.9 Hz) to C-1, C-9, and the signal of the carbonyl at δ_C_ 171.0. On the other hand, since Me-14 singlet gave cross peaks to the signals of the oxyquaternary carbon at δ_C_ 83.9 (C-6) and the quaternary sp^2^ carbon at δ_C_ 144.5 (C-5), the double bond was on C-5, and C-6 was oxygen bearing. This was corroborated by the HMBC cross peaks of the signal of the olefinic proton at δ_H_ 6.36, s (H-13) to C-4 and C-6. Moreover, the HMBC spectrum also exhibited a cross peak of H-13 signal to the signals of a conjugated carbonyl carbon at δ_C_ 161.3 (C-2′) and the quaternary sp^2^ carbon at δ_C_ 161.2 (C-4′). On the other hand, there were also HMBC cross peaks of another olefinic proton at δ_H_ 6.54, s (H-5′) to C-4′ and the signals of another two quaternary sp^2^ carbon at δ_C_ 101.1 (C-3′) and 157.2 (C-6′). Taken together the HMBC correlations, it was clear that the decahydronaphthalene ring system was fused, on C-5 and C-6, with *2H, 5H*-pyrano[4, 3-b]pyran-5-one ring system. The COSY and HMBC spectra also indicated the presence of the 3-substituted pyridine ring. That this pyridine ring was connected to the pyranone ring through C-3 of the former and C-6′ of the later was evidenced by the HMBC correlations of the H-5′ singlet to C-3″ (δ_C_ 127.4), as well as of the signal of H-2″ (δ_H_ 8.14, dt, *J* = 7.8, 2.4, 2.4 Hz) to C-6′. Literature search revealed that compound **6** was previously obtained by treatment of pyripyropene A with HCl under anhydrous condition [15]; however, there were neither reports of the ^1^H and ^13^C data nor other description of this compound. Since **6** is a new natural product, we have named it pyripyropene S. It is interesting to point out that **6** is the first natural pyripyropene that lacks a hydroxyl group on C-13.

**Table 3:** ^1^H and ^13^C NMR (CDCl_3_, 500.13 MHz and 125.8 MHz) and HMBC assignment for pyripyropene S (**6**).

Position	δ_C_, Type	δ_H_, (*J* in Hz)	COSY	HMBC
1	73.2, CH	4.79, dd (11.7, 4.6)	H-2	
2	23.2, CH_2_	1.99, m	H-1	
		1.76, m		
3	35.5, CH_2_	2.09, m	H-2	
4	38.8, C	-		
5	144.5, C	-		
6	83.9, C	-		
7	77.7, CH	5.23, dd (11.9, 5.4)	H-8	C-6
8	24.3, CH_2_	1.82, ddd (12.8, 5.1, 1.4)	H-7, H-9	
		1.61, m		
9	41.1, CH	1.73, brd (12.5)	H-8	
10	40.6, C	-		
11	64.7, CH_2_	3.75, d (11.9)		C-1, 9, CO (OAc-11)
		3.79, d (11.9)		
12	24.2, CH_3_	1.26, s		C-3, 4, 9
13	111.2, CH	6.36, s		C-4, 6, 4′
14	21.3, CH_3_	1.59,s		C-5, 6, 7
15	13.3, CH_3_	0.88, s		C-1, 10, 11
2′	161.3, C	-		
3′	101.1, C	-		
4′	161.2, C	-		
5′	98.6, CH	6.54, s		C-2′, 3′, 6′, 3″
6′	157.2, C	-		
2″	146.6, CH	9.02, brs	H-4″	C-3″, 4″, 6″
3″	127.4, C	-		
4″	133.1, CH	8.14, dt (7.8, 1.4, 1.4)	H-2″, 5″	C-6′, 2″, 6″
5″	123.8, CH	7.42, dd (8.0, 4.9)	H-4″, 6″	C-3″, 6″
6″	151.2, CH	8.68, brd (4.0)	H-5″	C-5″
OAc-1	170.4, CO	-		
	21.2, CH_3_	2.05, s		CO (OAc-1)
OAc-7	169.8, CO	-		
	21.2, CH_3_	2.17, s		CO (OAc-7)
OAc-11	171.0, CO	-		
	20.8, CH_3_	2.10, s		CO (OAc-11)

Compounds **1**, **2a**–**c** and **3**–**6** were tested for their antimicrobial activity against Gram positive (*Staphylococcus aureus* ATCC 25923 and *Bacillus subtilis* ATCC 6633) and Gram negative (*Escherichia coli* ATCC 25922 and *Pseudomonas aeruginosa* ATCC 27853) bacteria, *Candida albicans* ATCC 10231, and multidrug-resistant isolates from the environment. All the compounds tested exhibited neither antibacterial nor antifungal activities, *i.e.*, their MIC values were found to be higher than 256 µg/mL. Like chevalone C, chevalone E (**3**) does not possess the structural requirements for the antibacterial activity of this group of meroditerpenes, *i.e*., the presence of the β-acetoxyl group on C-3 and the presence of a free 4-hydroxy-6-methyl-2*H*-pyran-2-one ring on C-15 [9]. Therefore, it is not surprising that chevalone E (**3**) did not exhibit significant antibacterial activity. The fact that chevalone C did not show significant antibacterial activity but demonstrated synergistic effect with the antibiotics against three multidrug-resistant isolates [9] led us explore if some of these compounds could possibly have synergistic effects with antibiotics, *i.e.*, by using a disc diffusion method to assess if, in combination with antibiotics, they could cause an increase in the growth inhibition of multidrug-resistant strains. The results (Table 4) showed that no synergistic effects were observed between the tested compounds and antibiotics for multidrug-resistant *E. coli* and *E. faecalis*; however chevalone E (**3**) was found to exhibit potential synergy with oxacillin and ampicillin against the MRSA strain.

**Table 4:** Antibacterial efficacy of combined effect of antibiotics with the compounds (15 µg/disc) against three multidrug-resistant isolates, using the disc diffusion method.

Compound	*E. coli* G1				*S. aureus* B1			*E. faecalis* W1		
	Antibiotics									
	CIP	AMP	CTX	S	OX	AMP	CIP	VA	AMP	E
**1**	−	−	−	−	−	−	−	−	−	−
**2a**	−	−	−	−	−	++	−	−	−	−
**2b**	−	−	−	−	−	++	−	−	−	−
**2c**				−	−	++	−	−	−	−
**3**	−	−	−	−	+++	+++	−	−	−	−
**4**			−	−		++	−	−	−	−
**5**	−	−	−	−	−	++	−	−	−	−
**6**	−	−	−	−	−	++	−	−	−	−

(−) noneffective; (+) slight efficacy—halo of inhibition or additional increase in the halo of 1 to 2.5 mm around the disc; (++) moderate efficacy—from >2.5 to 5 mm; (+++) good efficacy—from >5 to 8 mm; CIP: ciprofloxacin; AMP: ampicillin; CTX: cefotaxime; S: streptomycin; OX: oxacillin; VA: vancomycin; E: Erythromycin.

In order to verify if the synergism occurred with both antibiotics or with either of them, the checkerboard method was carried out. The results, represented by the fractional inhibitory concentration (FIC) index, shown in Table 5, confirmed the synergy between chevalone E (**3**) and oxacillin, and not between chevalone E (**3**) and ampicillin. It is interesting to note that while chevalone E (**3**) shows synergism with oxacillin against the MRSA isolate, the structurally related meroditerpene aszonapyrone exhibited synergism only with vancomycin against the VRE isolate, and not with oxacillin against the MRSA strain [9].

**Table 5:** MIC values of chevalone E (**3**) in combination with oxacillin or ampicillin, and the respective FIC index obtained against a MRSA (*S. aureus* B1) using the checkerboard method.

Strain	MIC (µg/mL)				
*S. aureus* B1	**3** alone	OX alone	**3** with OX	OX with **3**	FIC index
	˃1024	128	64	16	˂0.188 *
*S. aureus* B1	**3** alone	AMP alone	**3** with AMP	AMP with **3**	FIC index
	˃1024	128	˃512	128	˃1.5

* FIC index < 0.5 indicates synergy.

## 3. Experimental Section

### 3.1. General Procedures

Melting points were determined on a Bock monoscope and are uncorrected. Optical rotations were determined on an ADP410 Polarimeter (Bellingham + Syanley Ltd., Tunbridge Wells, Kent, UK). Infrared spectra were recorded on an ATT Mattson Genesis Series FTIR™ using WinFIRST Software. ^1^H and ^13^C NMR spectra were recorded at ambient temperature on a Bruker AMC instrument (Bruker Biosciences Corporation, Billerica, MA, USA) operating at 500.13 and 125.8 MHz, respectively. High resolution mass spectra were measured with a Waters Xevo QToF mass spectrometer (Waters Corporations, Milford, MA, USA) coupled to a Waters Aquity UPLC system. A Merck silica gel GF_254_ was used for preparative TLC, and a Merck Si gel 60 (0.2–0.5 mm) was used for analytical chromatography.

### 3.2. Extraction and Isolation

The strain KUFA0013 was isolated from the marine sponge *Rhabdermia* sp., which was collected from the coral reef of the Similan Islands, Phang Nga Province, Thailand, by scuba diving at 10 m depth, in April 2010, and the sponge was identified by J. Buaruang (Division of Environmental Science, Faculty of Science, Ramkhamhaeng University, Bangkok 10240, Thailand). Briefly, after rinsing with sterile sea water, the sponge was dried on sterile filter paper and cut into small pieces (5 × 5 mm) and placed on the plates containing malt extract agar [MEA, 30 g of malt extract powder (Himedia, Mumbai, India), 15 g of bacto agar, distilled water 300 mL, sea water 700 mL and adjusted to the final pH at 5.5] with 70% sea water and incubated at 28 °C under 12 h light/12 h dark cycle for seven days. The fungus was identified by one of us (T. Dethoup), by morphological features, including characteristic of ascospores, conidiogenesis and colonies, as well as by DNA sequence analysis of the calmodulin gene described by the previous report [16] (GenBank Accession No. KC 920702). Since the sequence was not identical to that deposited at GenBank, the strain was not identified at species level. The pure cultures were deposited as KUFA0013 at the Department of Plant Pathology, Faculty of Agriculture, Kasetsart University, Bangkok, Thailand. *A. similanensis* (KUFA 0013) was cultured for one week in five 90 mm Petri dishes (i.d. 90 mm) containing 25 mL of MEA with 70% sea water per dish. Thirty 1000 mL Erlenmeyer flasks, each containing white rice (200 g), water (30 mL) and sea water (70 mL), were autoclaved at 121 °C for 15 min, inoculated with ten mycelia plugs of the fungus and incubated at 28 °C for 30 days. The moldy rice was macerated in ethyl acetate (7 L total) for seven days and then filtered by filter paper. The two layers were separated using a separatory funnel, and the ethyl acetate solution was evaporated under reduced pressure to yield 97 g of crude ethyl acetate extract that was dissolved in 500 mL of a 4:1 mixture of EtOAc and CHCl_3_, and then washed with 5% NaHCO_3_ aqueous solution (2 × 300 mL) and H_2_O (3 × 300 mL). The organic layer was dried with anhydrous Na_2_SO_4_, filtered and evaporated under reduced pressure to give 75 g of crude extract, which was applied on a column chromatography of silica gel (640 g) and eluted with mixtures of CHCl_3_–petrol and CHCl_3_–Me_2_CO, 250 mL fractions were collected as follows: Frs 1–18 (CHCl_3_–petrol, 3:7), 19–53 (CHCl_3_–petrol, 1:1), 54–114 (CHCl_3_–petrol, 7:3), 115–215 (CHCl_3_–petrol, 9:1), 216–395 (CHCl_3_–Me_2_CO, 9:1), 396–443 (CHCl_3_–Me_2_CO, 7:3). Frs 185–196 were combined (654 mg) and purified by TLC (Si gel, CHCl_3_:Me_2_CO:HCO_2_H, 97:3:0.01) to give 4 mg of **1**. Frs 197–221 were combined (1.16 g) and crystallized in a mixture of petrol and CHCl_3_ to give additional 107.6 mg of yellow solid which was further purified by TLC (Si gel, CHCl_3_:Me_2_CO:HCO_2_H, 98:2:0.01) to give 2.5 mg of **1**. Fr 222 (8.06 g) was recrystallized in a mixture of CHCl_3_ and Me_2_CO to give 238 mg of white precipitate, which was further purified by TLC (Si gel, CHCl_3_:Me_2_CO:HCO_2_H, 97:3:0.01) to give **2b** (32.7 mg), **4** (4.6 mg) and chevalone B (4.6 mg). The mother liquor was further purified by TLC (Si gel, CHCl_3_:Me_2_CO:HCO_2_H, 97:3:0.01) to give **2b** (41.0 mg), **4** (7.2 mg) and chevalone B (70.8 mg). The mother liquor of frs 197–221 and fr 222, and frs 223–224 were combined (9.18 g), applied on the Si gel column (58 g), and eluted with mixtures of petrol–CHCl_3_ and CHCl_3_–Me_2_CO, wherein 100 mL sfrs were collected as follows: sfrs 1-59 (petrol–CHCl_3_, 3:7), 60–69 (petrol–CHCl_3_, 1:9), 70–76 (CHCl_3_–Me_2_CO, 9:1). Sfrs 11–22 were combined (1.97 g) and crystallized in a mixture of petrol and CHCl_3_ to give additional 16.4 mg of **1**. Sfrs 29–42 were combined (468 mg) and crystallized in a mixture of petrol and CHCl_3_ to give additional 92.1 mg of **2b**. Frs 225–228 were combined (446 mg) and crystallized in a mixture of petrol and CHCl_3_ to give 63 mg of a precipitate which was further purified by TLC (Si gel, CHCl_3_:Me_2_CO:HCO_2_H, 97:3:0.01) to give **2b** (35.8 mg) and **2a** (35.4 mg). The mother liquor of frs 225–228 and frs 229–330 were combined and chromatographed on a Si gel column (33 g) and eluted with mixtures of petrol–CHCl_3_ and CHCl_3_–Me_2_CO, wherein 100 mL sub-fractions were collected as follows: sfrs 1–49 (petrol–CHCl_3_, 3:7), 50–64 (petrol–CHCl_3_, 1:9), 65–77 (CHCl_3_–Me_2_CO, 9:1). Subfrs 4–5 were combined and recrystallized in a mixture of petrol and CHCl_3_ to give **1** (2.4 mg). Sfrs 6–10 were combined (160 mg) and recrystallized in a mixture of petrol and CHCl_3_ to give **2c** (7.6 mg). Sfrs 11–16 were combined (108 mg) and recrystallized in a mixture of petrol to give **2b** (5 mg). Sfrs 27-33 were combined (206 mg) and purified by TLC (Si gel, CHCl_3_: Me_2_CO. 93:7) to give *p*-hydroxybenzaldehyde (36 mg). Frs 231–247 were combined (6.7 g) and recrystallized in a mixture of petrol and Me_2_CO to give 1.39 g of chevalone C. Frs 272–294 were combined (1.54 g) and crystallized in a mixture of petrol and Me_2_CO to yield **5** (265 mg). Frs 328-335 were combined (296 mg) and applied on a column of Sephadex LH-20 (22 g) and eluted with a mixture of CHCl_3_–MeOH (9:1) to give **3** (11.2 mg). Frs 354–398 were combined (1.14 g) and purified by TLC (Si gel, CHCl_3_:MeOH:HCO_2_H, 95:5:0.01) to give **6** (27.3 mg).

#### 3.2.1. Similanpyrone A (**1**)

White solid, Mp 322–323 °C (petrol/CHCl_3_); UV (CHCl_3_) λ_max_ (log ε) 240 (4.31), 269 (4.31), 295 (3.95), 333 (4.23), 358 (4.16) nm; IR (KBr) ν_max_ 3446, 3010, 2923, 2851, 1748, 1698, 1658, 1634, 1464, 1177, 1151 cm^−1^; ^1^H and ^13^C NMR (Table 1); HRESIMS *m/z* 245.0455 (M + H)^+^ (calculated for C_13_H_9_O_5_, 245.0450).

#### 3.2.2. Similanpyrone B (**2b**)

White crystals, Mp 162–163 °C (petrol/CHCl_3_); UV (CHCl_3_) λ_max_ (log ε) 240 (4.35), 277 (3.51), 330 (3.46) nm; IR (KBr) ν_max_ 3243, 3160, 2923, 2851, 1677, 1634, 1617, 1585, 1571, 1455, 1256, 1154, 1110 cm^−1^; ^1^H and ^13^C NMR (Table 2); HRESIMS *m/z* 207.0658 (M + H)^+^ (calculated for C_11_H_11_O_4_, 207.0657).

#### 3.2.3. Chevalone E (**3**)

White crystals, Mp 262–263 °C (petrol/CHCl_3_); [α]_D_^20^ −146.3° (*c* 0.04, CHCl_3_); IR (KBr) ν_max_ 3300, 3016, 2979, 2950, 2871, 1664, 1607, 1570, 1444, 1288 cm^−1^; ^1^H NMR (CDCl_3_, 500.13 MHz) δ 5.99 (1H, s, H-18), 3.21 (1H, d, *J* = 11.1, 5.0, H-3), 2.55 (1H, dd, *J* = 16.4, 4.9, H_2_-15), 2.20 (3H, s, H_3_-20), 2.15 (1H, m, H_2_-15), 2.14 (1H, m, H_2_-12), 1.90 (1H, dt, *J* = 12.8, 3, H_2_-7), 1.74 (1H, m, H_2_-11), 1.73 (1H, m, H_2_-1), 1.71 (1H, m, H_2_-12), 1.64 (2H, m, H_2_-2), 1.59 (1H, m, H_2_-6), 1.50 (1H, dd, *J* = 12.5, 4.7, H-14), 1.45 (1H, m, H_2_-6), 1.36 (1H, m, H_2_-11), 1.28 (3H, s, H_3_-26), 1.05 (1H, m, H_2_-7), 1.00 (1H, m, H_2_-1), 0.98 (3H, s, H_3_-23), 0.93 (1H, brd, *J* = 13.2, H-9), 0.89 (3H, s, H_3_-25), 0.84 (3H, s, H_3_-24), 0.78 (1H, brd, *J* = 11-6, H-5), 0.78 (3H, s, H_3_-22); ^13^C NMR (CDCl_3_, 125.8 MHz) δ 180.6 (CO, C-17), 162 (C, C-16), 160.5 (C, C-21), 111.9 (CH, C-18), 98.5 (C, C-19), 84.3 (C, C-13), 78.7 (CH, C-3), 60.3 (CH, C-9), 55.3 (CH, C-5), 52.3 (CH, C-14), 41.1 (CH_2_, C-7), 40.1 (CH_2_, C-12), 38.9 (C, C-4), 38.4 (CH_2_, C-1), 37.3 (C, C-8), 37.1 (C, C-10), 28.0 (CH_3_, C-23), 27.2 (CH_2_, C-2), 20.5 (CH_3_, C-26), 19.2 (CH_3_, C-20), 18.7 (CH_2_, C-11), 17.9 (CH_2_, C-6), 16.4 (CH_3_, C-24), 16.1 (CH_3_, C-25), 15.4 (CH_3_, C-22), 15.2 (CH_3_, C-15); HRESIMS *m/z* 415.2851 (M + H)^+^ (calculated for C_26_H_39_O_4_, 415.2848).

#### 3.2.4. Pyripyropene S (**6**)

Yellow viscous liquid, [α]_D_^20^ +116.3 (*c* 0.04, CHCl_3_); IR (KBr*)* ν_max_ *2*923, 2851, 1742, 1671, 1624, 1586, 1508, 1465, 1374, 1242, 1043 cm^−1^; ^1^H and ^13^C NMR (Table 3); HRESIMS *m/z* 566.2415 (M + H)^+^ (calculated for C_31_H_36_NO_9_, 566.2390).

### 3.3. X-ray Crystal Structure of Chevalone E (**3**)

Crystals suitable for X-ray diffraction were obtained by slow evaporation of a solution in petroleum ether/chloroform. They were orthorhombic, space group P2_1_2_1_2_1_, cell volume 2310.9(1) Å^3^ and unit cell dimensions *a* = 8.2325(2) Å, *b* =11.3341(3) Å and *c* = 24.7665(6) Å. Diffraction data were collected at 293 K with a Gemini PX Ultra equipped with CuK_α_ radiation (λ = 1.54184 Å). The structures were solved by direct methods using SHELXS-97 and refined with SHELXL-97. Carbon, oxygen and nitrogen atoms were refined anisotropically. Hydrogen atoms were refined freely with isotropic displacement parameters. The refinement converged to *R* (all data) = 6.38% and *wR_2_* (all data) = 10.21%. Towards the end of refinement the absolute structure parameter *x* (Flack *x* parameter) was refined at the same time as all other parameters, using the TWIN instruction with the default matrix R = (−1 0 0, 0 −1 0, 0 0 −1) and BASF with one parameter (*x*), to reach the final value of *x* = 0.0 (3). The inverted structure, obtained with the instruction MOVE 1 1 1 −1, yielded *x* = 1.5 (3). Tables containing the final fractional coordinates, temperature parameters, bond distances, and bond angles were deposited with the Cambridge Crystallographic Data Centre: CCDC reference number 1002416.

### 3.4. Antimicrobial Activity Assays

#### 3.4.1. Bacterial Strains

For the antimicrobial assays, the compounds were tested against: bacterial reference strains (*Staphylococcus aureus* ATCC 25923, *Bacillus subtilis* ATCC 6633, *Escherichia coli* ATCC 25922 and *Pseudomonas aeruginosa* ATCC 27853), *Candida albicans* ATCC 10231 and multidrug-resistant bacteria isolated from the environment, *S. aureus* B1 (isolated from public bus), *Enterococcus faecalis* W1 (isolated from river water) and *E. coli* G1 (isolated from seagull feces). Bacteria were grown in Mueller-Hinton agar (MH-BioKar diagnostics, Allonne, France) from stock cultures, while *C. albicans* was grown in Sabouraud dextrose agar (SAB-BioKar diagnostics, Allonne, France). MH and SAB plates were incubated at 37 °C prior to obtain fresh cultures for each *in vitro* bioassay.

#### 3.4.2. Determination of Minimum Inhibitory and Bactericidal/Fungal Concentrations

The minimum inhibitory concentrations (MIC) of the compounds were determined using a broth microdilution technique, following the recommendations of the Clinical and Laboratory Standards Institute [17]. Stock solutions of 10 mg/mL, prepared in dimethylsulfoxide (DMSO-Applichem GmbH, Darmstadt, Germany), were serially diluted in Mueller-Hinton broth (MHB-BioKar diagnostics, Allonne, France) to achieve in-test concentrations ranging from 2 to 256 µg/mL. Each bacterial inoculum was prepared in MHB, while *C. albicans* inoculum was prepared in RPMI-1640 with l-glutamine, with MOPS and without NaHCO_3_ (Lonza, Walkersville, MD, USA). All inocula were standardized in order obtain a concentration of 5 × 10^5^ CFU/mL in each inoculated well of the microtiter plate. The concentration of DMSO in the highest in-test concentration did not affect the microbial growth. The MIC was defined as the lowest concentration of compound that has inhibited the visible growth. 

#### 3.4.3. Synergistic Studies

##### 3.4.3.1. Screening of Combined Effect between the Compounds and Antibiotics

A screening susceptibility test to assess the combined effect between the compounds and antibiotics was conducted using the disc diffusion method on MH, according to the procedure already described by Gomes *et al.* [9].

##### 3.4.3.2. Synergy Test: Checkerboard Method

Based on the results of the previous assay, potential synergy between **3** and oxacillin or ampicillin (Sigma-Aldrich, St. Louis, MO, USA) was checked using a broth microdilution checkerboard method and tested in MRSA isolate (*S. aureus* B1), as has been already described [9]. Two independent experiments in duplicate were performed. The fractional inhibitory concentration (FIC) was calculated as follows: FIC of drug A (FIC A) = MIC of drug A in combination/MIC of drug A alone, and FIC of drug B (FIC B) = MIC of drug B in combination/MIC of drug B alone. The FIC index (ΣFIC), calculated as the sum of each FIC, was interpreted as follows: ΣFIC ≤ 0.5, synergy; 0.5 < ΣFIC ≤ 4, no interaction; 4 < ΣFIC, antagonism [18].

## 4. Conclusions

Although several analogs of chevalone have been reported from several members of the genus *Aspergillus*, this is the first report of isolation of isocoumarin derivatives from a member of this genus. The synergism of chevalone E with the antibiotic oxacillin against MRSA can be considered relevant for anti-infective marine natural products research.

## Supplementary Files

Supplementary File 1
