# Supplementary material for: New Isocoumarin Derivatives and Meroterpenoids from the Marine Sponge-Associated Fungus Aspergillus similanensis sp. nov. KUFA 0013
Source: Mar Drugs. 2014 Oct 13;12(10):5160–73. doi: 10.3390/md12105160 (PMC4210891; doi:10.3390/md12105160)
Supplement: Supplementary File 1 [file marinedrugs-12-05160-s001.pdf]

Supplementary Information

Figure S1. ^{1}H NMR spectrum of compound **1** ($CDCl_3$, 500.13 MHz).

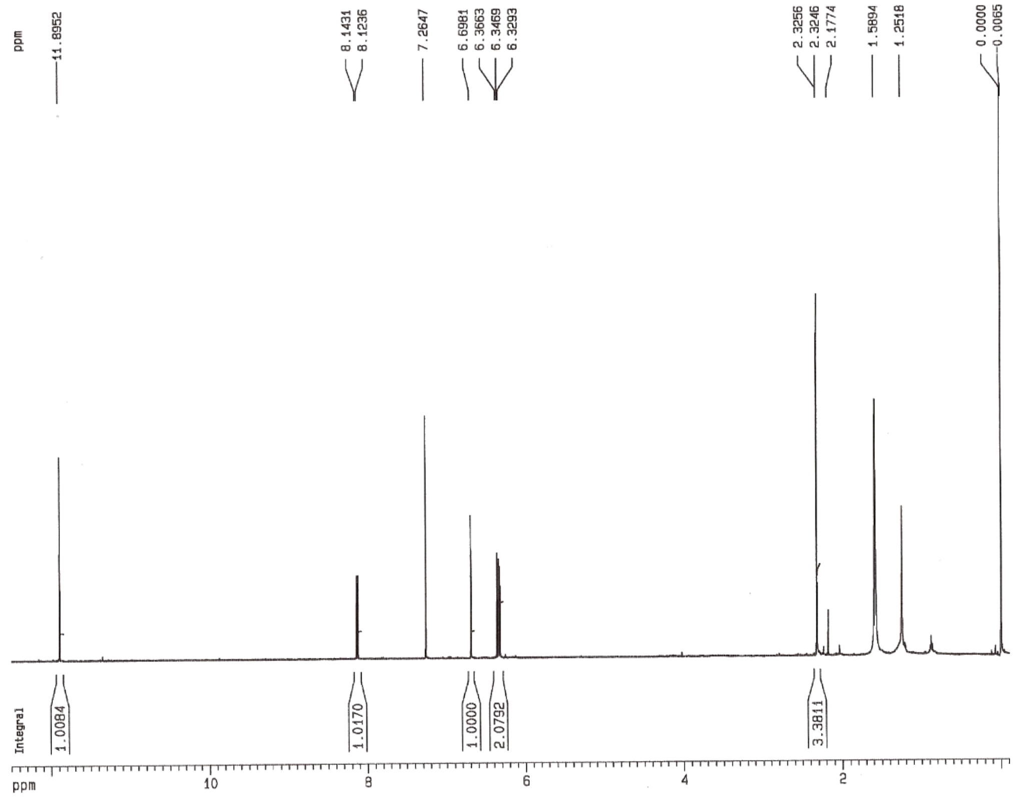

Figure S2. ^{13}C NMR spectrum of compound **1** ($CDCl_3$, 125.8 MHz).

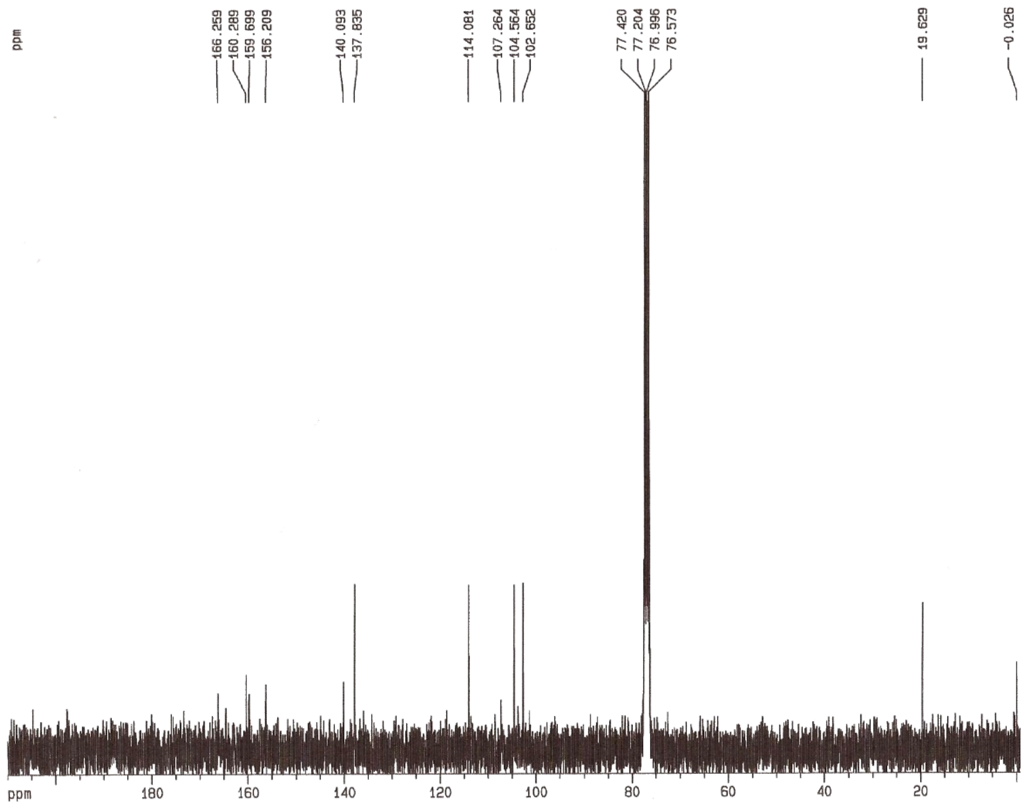

Figure S3. COSY spectrum of compound **1** ($CDCl_3$, 500.13 MHz MHz).

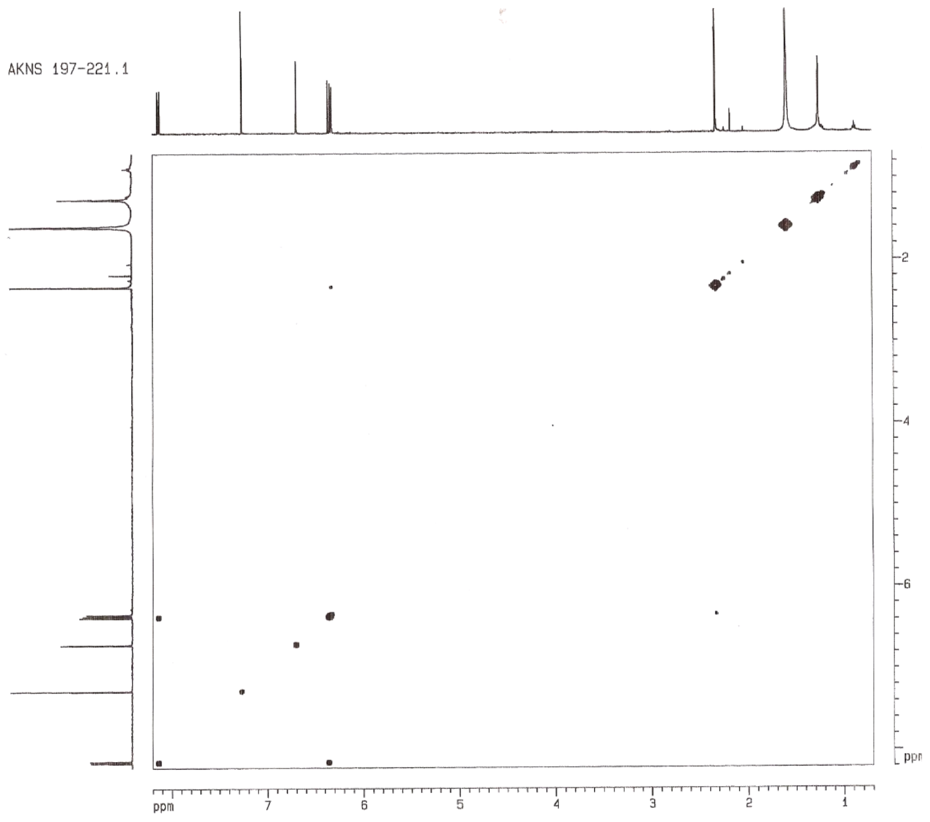

Figure S4. HSQC spectrum of compound **1** ($CDCl_3$, 500.13 MHz MHz).

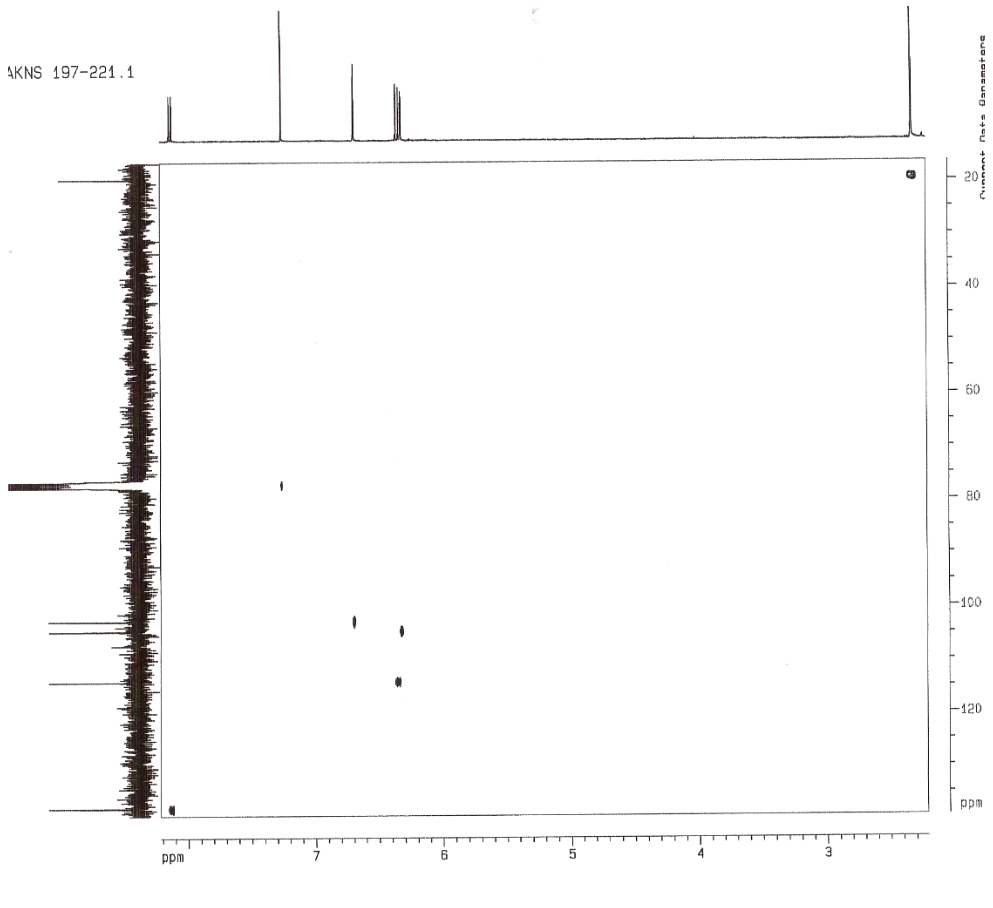

Figure S5. HMBC spectrum of compound **1** ($CDCl_3$, 500.13 MHz MHz).

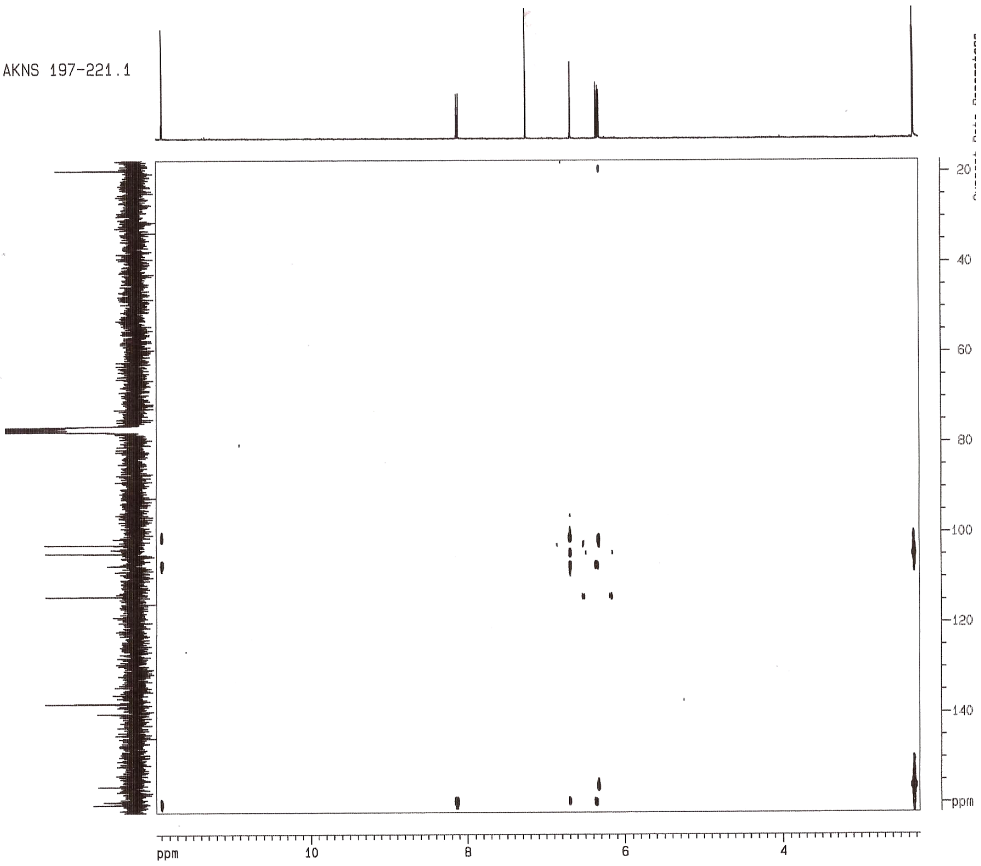

Figure S6. ^{1}H NMR spectrum of compound **2b** ($CDCl_3$, 500.13 MHz).

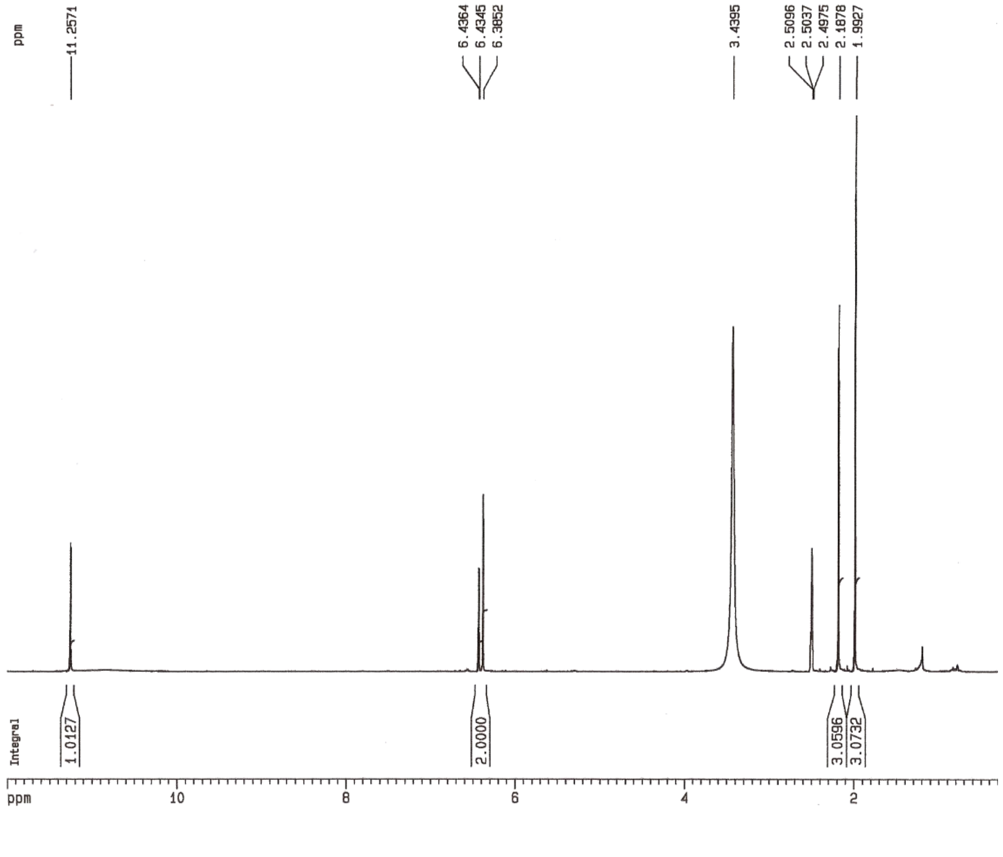

Figure S7. ^{13}C NMR spectrum of compound **2b** ($CDCl_3$, 125.8 MHz).

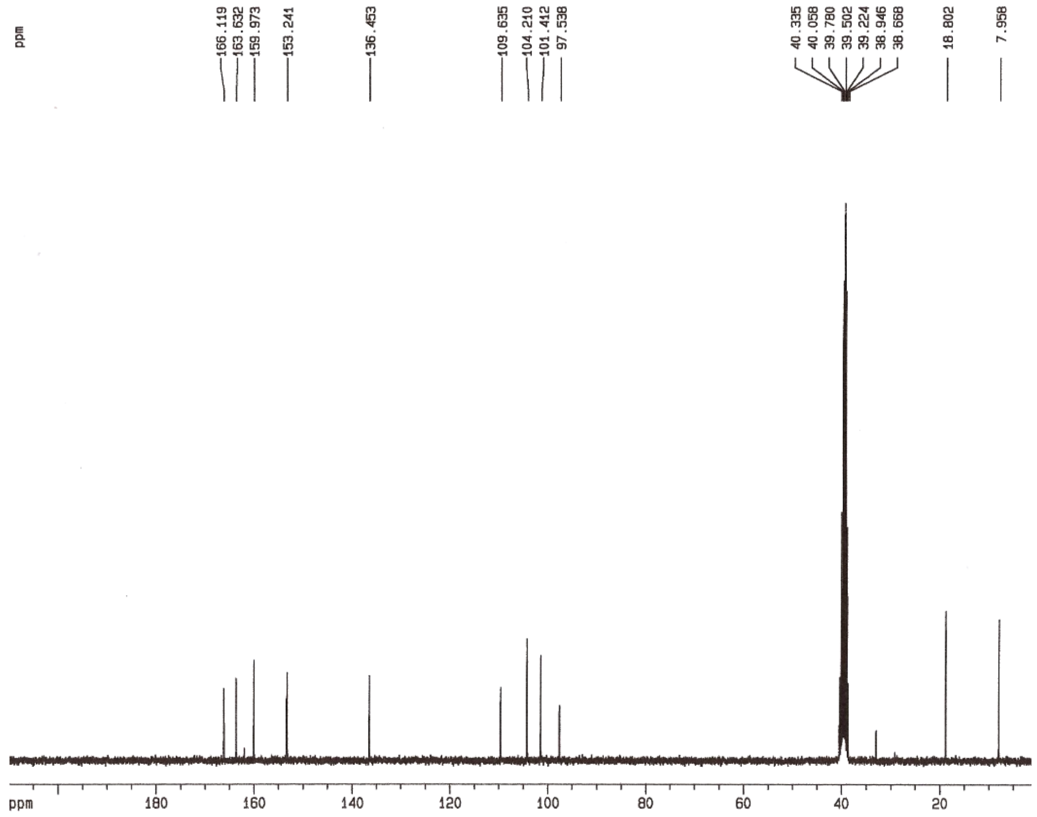

Figure S8. ^{1}H NMR spectrum of compound **3** ($CDCl_3$, 500.13 MHz).

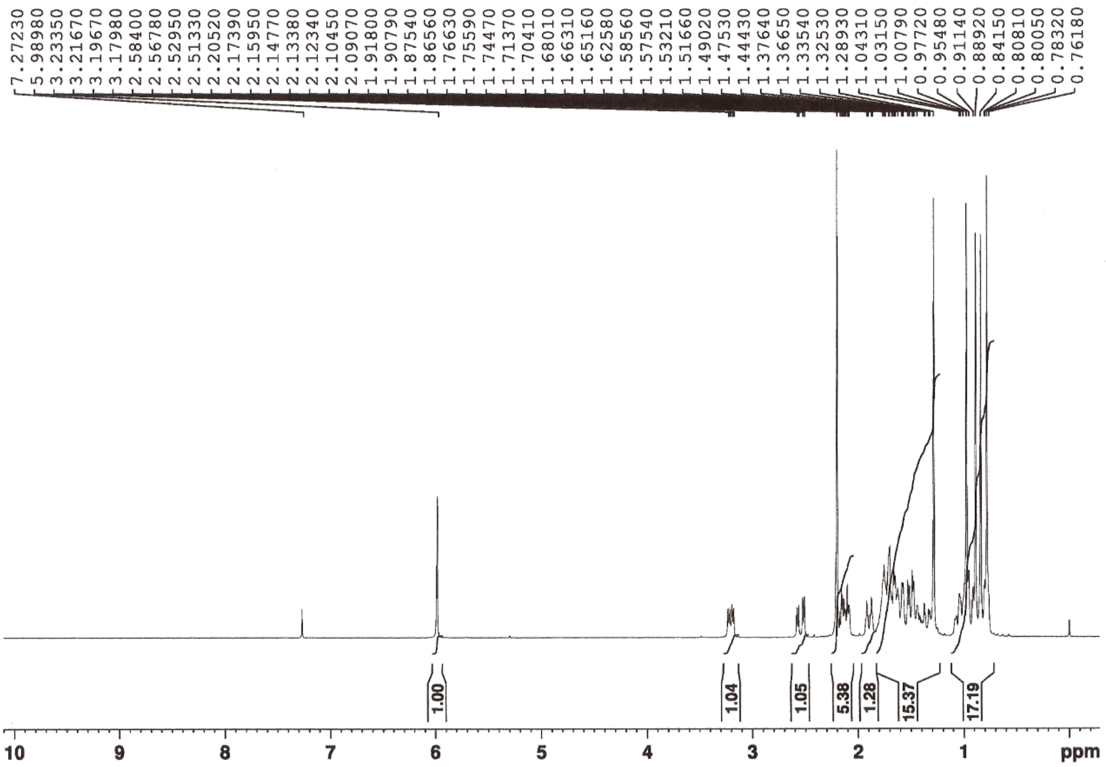

Figure S9. ^{13}C NMR spectrum of compound **3** ($CDCl_3$, 125.8 MHz).

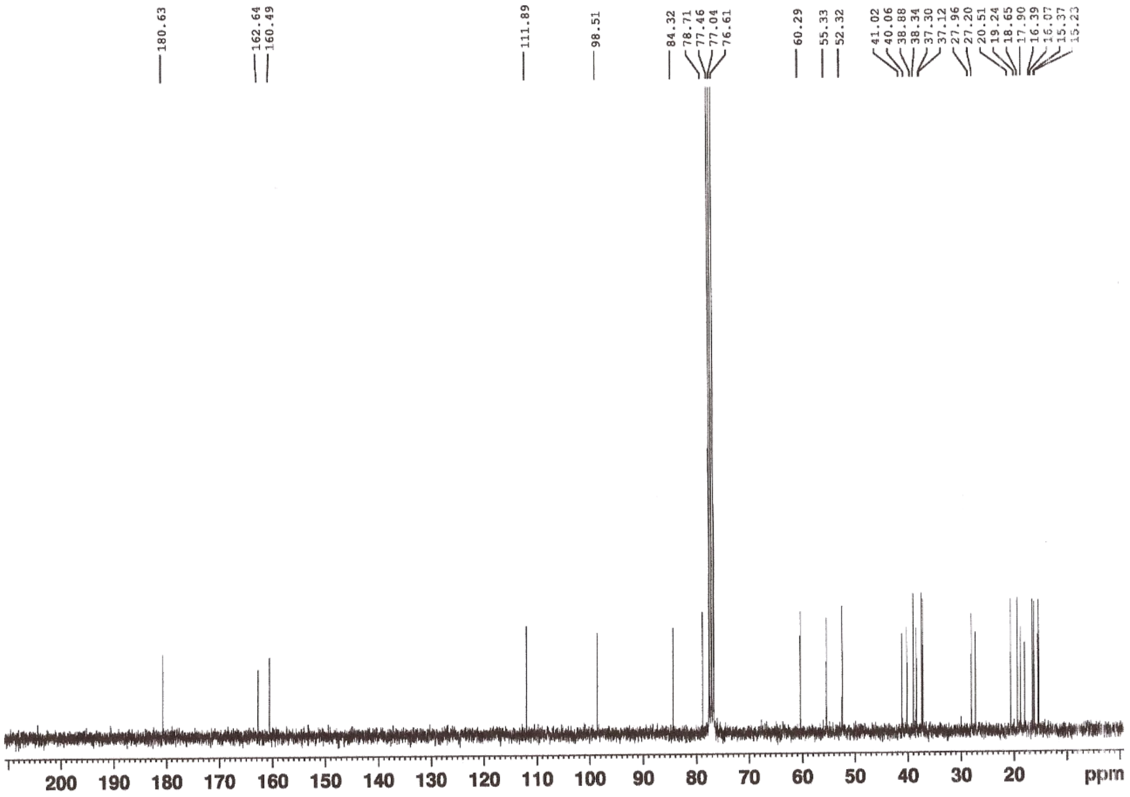

Figure S10. ^{1}H NMR spectrum of compound **6** ($CDCl_3$, 500.13 MHz).

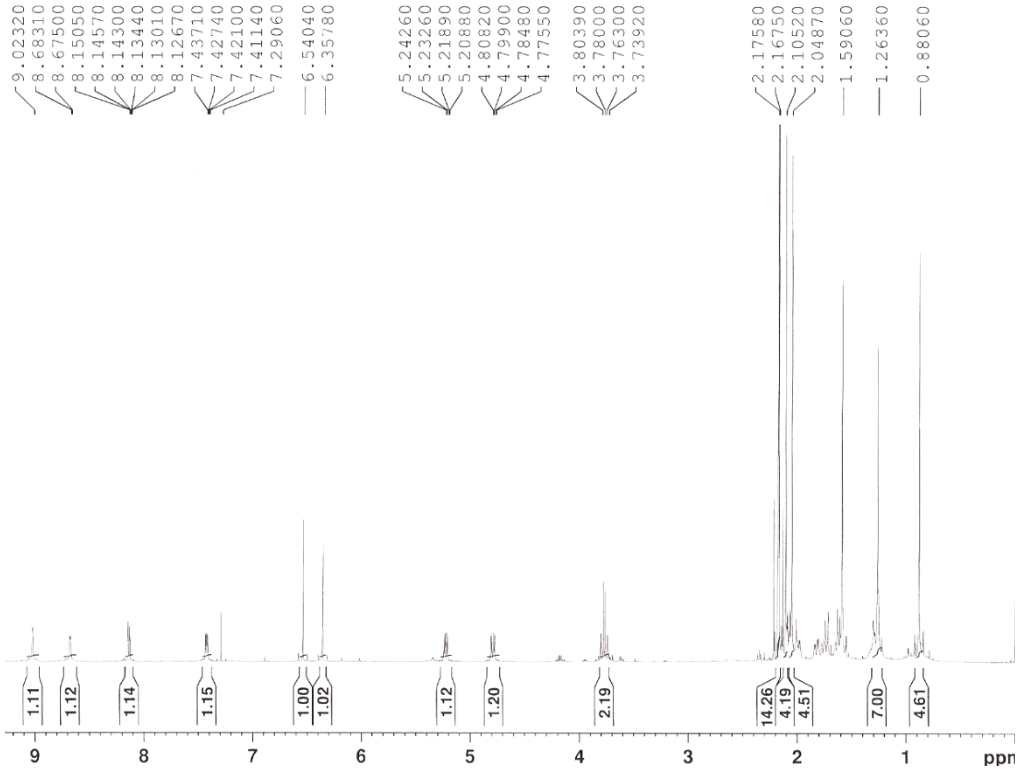

Figure S11. ^{13}C NMR spectrum of compound **6** ($CDCl_3$, 125.8 MHz).

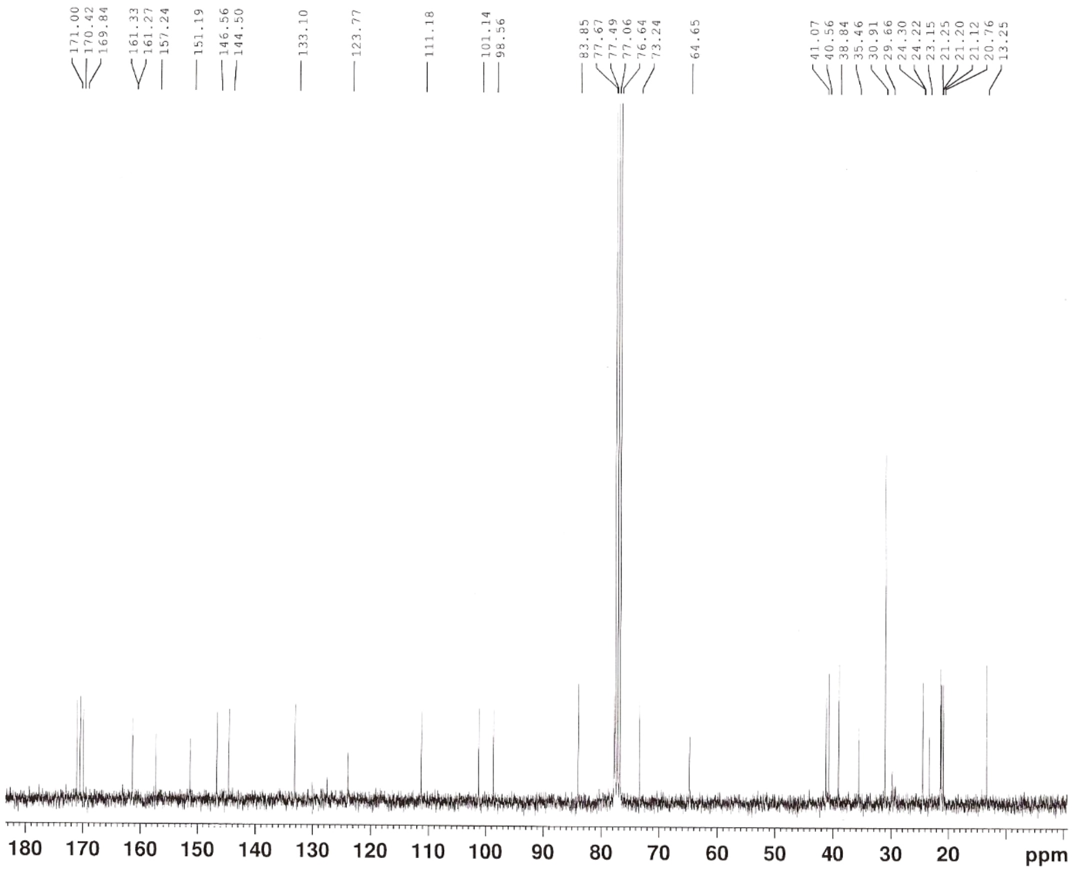

© 2014 by the authors; licensee MDPI, Basel, Switzerland. This article is an open access article distributed under the terms and conditions of the Creative Commons Attribution license (http://creativecommons.org/licenses/by/3.0/).
